# Accuracy of Peripheral Arterial Disease Registers in UK General Practice: Case-Control Study

**DOI:** 10.1177/2150132720946148

**Published:** 2020-09-22

**Authors:** Daniel Kyle, Luke Boylan, Lesley Wilson, Shona Haining, Crispian Oates, Andrew Sims, Ina Guri, John Allen, Scott Wilkes, Gerry Stansby

**Affiliations:** 1 Freeman Hospital, The Newcastle upon Tyne NHS Hospitals Foundation Trust, Newcastle upon Tyne, UK; 2 University of Newcastle, Newcastle upon Tyne, UK; 3 North of England Commissioning Support (NECS), Durham, UK; 4 University of Sunderland, Sunderland, Tyne and Wear, UK

**Keywords:** Peripheral arterial disease, screening, primary care, duplex ultrasound scanning

## Abstract

**Background:**

Approximately 20% of the UK population aged 55 to 75 years have evidence of peripheral arterial disease (PAD). PAD affects quality of life and life expectancy if not appropriately diagnosed and managed. At risk patients require accurate diagnosis to ensure optimal treatment to slow disease progression and minimize adverse outcomes.

**Aim:**

To assess the accuracy of general practice (GP) registration of the diagnosis of peripheral arterial disease (PAD).

**Design and Setting:**

An observational analytic case-control study. As part of a National Institute for Health Research–funded (ISRCTN13301188) project assessing novel diagnostic methods set in GP practice.

**Methods:**

A total of 125 patients registered as having PAD and 125 age- and sex-matched controls were recruited from 15 general practices across North East England. The register was then assessed for accuracy of diagnosis. Duplex vascular ultrasound scanning (DUS) undertaken by vascular scientists was used as the gold standard reference for PAD.

**Results:**

The PAD register had a sensitivity of 86% (95% CI 77%-92%) and specificity of 74% (95% CI 67%-81%) when compared with DUS. The positive predictive value, however, was 69.6% (95% CI 63%-75%) and negative predictive value 88.8% (95% CI 82%-92%). The overall diagnostic effectiveness of the PAD register was 79.2% (95% CI 73%-84%).

**Conclusion:**

This analysis indicates that while PAD is detected with reasonable sensitivity in primary care, many patients registered with a diagnosis of PAD lacked DUS-proven disease. Improved approaches to the objective diagnosis of PAD may improve diagnosis and management of PAD in primary care.

## Introduction

Peripheral arterial disease (PAD) is a manifestation of atherosclerosis in the peripheral arteries, resulting in reduced lower limb tissue perfusion.^1^ There is limited evidence on the prevalence of PAD; however, about 15% to 20% of the UK population aged 55 to 75 years have evidence of lower extremity PAD on objective testing.^1,2^ To put this into perspective, a general practice (GP) with 20 000 registered patients would expect to see 40 newly diagnosed patients with PAD each year.^3^ PAD poses a significant disease burden carrying a poor prognosis once well established; partly owing to its association with cerebral and coronary artery disease, which is concomitant in 65% of PAD patients.^4^ The most common symptom is intermittent claudication (IC), which causes predictable and repeatable ischemic pain in the calf on exertion due to reduced tissue perfusion relieved by rest. However, many patients are asymptomatic with IC only present in approximately 4.5% of patients aged 55 to 74 years.^2^ As the disease progresses a patient may then present with ischemic pain at rest, tissue gangrene, ulceration, or necrosis collectively known as critical limb ischemia (CLI). CLI has a 50% to 60% 5-year survival and is associated with minor and major amputations.^5^

The prevalence of PAD in the population depends on whether the diagnosis is made on clinical grounds only instead of using objective methods such as ankle brachial pressure index (ABPI). It also depends on the definition of PAD used and the population studied.^6^ The standard test recommended by NICE (National Institute for Health and Care Excellence) for confirmation of a diagnosis of PAD in GP is the ABPI. ABPI is noninvasive, inexpensive, and has reasonable inter- and intraobserver reliability,^7^ though it is not without a number of limitations in assessing PAD in primary care.^8^ It is a clinical imperative that “at-risk” patients are accurately diagnosed to ensure optimal, early treatment to reduce risk of disease progression, while minimizing unnecessary treatment of patients without proven disease.

Assessment of PAD in primary care classically relies on a combination of clinical assessment, ABPI measurements, and clinical risk stratifying tools.^6^ Previous work has assessed these components individually in their clinical utility at assessing peripheral vascular disease in primary care.^6^ This study examines the GP’s assessment of peripheral arterial disease as a whole and uses duplex ultrasound scanning as a gold standard investigation for comparison given its very high correlation with digital subtraction angiography in identifying lower limb PAD.^9^ In doing so, this work investigates the accuracy of PAD registers and highlights a need for improved assessment of PAD in primary care.

## Methods

### Design

As part of a diagnostic evaluation for a new diagnostic test in PAD (NOTEPAD Study), patients registered with PAD and control group patients with no registered diagnosis of PAD were recruited over a 16-month period between May 2015 and September 2016 from 15 different GPs across the North East of England. The control group patients were matched for age and sex.

Duplex ultrasound scanning (DUS) undertaken by appropriately trained vascular scientists formed the comparative standard for PAD diagnosis for the wider study. The vascular scientists were blinded to the registry status of the patient being assessed. All patients included had documented ABPI values reassessed as part of this trial with additional multisite photoplethysmography (MPPG) measurements recorded as part of a wider National Institute for Health Research (NIHR)–funded (ISRCTN13301188) NOTEPAD project investigating novel diagnostic methods of assessing PAD. The MPPG evaluation will be analyzed and published separately.

### Setting

All measurements and patient information were recorded within the participating GP, including DUS assessment. This was to reduce biasing participants who were unable to attend a tertiary centre for assessment and to investigate new methods of PAD assessment in primary care.

### Participants

Those eligible were identified by the GP and invited to attend for initial screening. Patients included were all older than 45 years. Baseline demographics and comorbidities were recorded by a vascular research nurse specialist who was blinded to the registry status of the patient. At this point, patients were informed of the purpose of their participation as part of an informed consent process. Control group patients matched for age and sex were then identified and invited for participation across the different sites. Patients were recruited over a 1-year period. Participants were coded using the GP site, followed by the number that patient corresponded to in chronological order of recruitment from that practice. For example, the second patient at GP site 2 was encoded 2-002. No information regarding the vascular status of that patient was included in the coding structure so as to maintain the blinded status of the individual being assessed by the vascular scientist.

### Statistical Analysis

The R programming language was used for data cleaning and analysis.^10^ Pearson’s chi-square test with Yates’ continuity correction was applied to categorical data to assess independence and the Welch 2-sample *t* test was used to assess significant difference between the means of the 2 groups.

### Duplex Vascular Ultrasound Scan

All patients underwent bilateral DUS in the primary care setting. Each scan was performed by a trained vascular scientist. The vascular scientist was blinded to the registry status of the patient and received only the coded participant number presenting for assessment. They did not have access to the patient’s GP records and produced and submitted their reports as soon as the vascular assessment had been performed. Patients were assessed while lying supine and the limb was scanned from the groin to the ankle. Waveforms and velocities were measured to gauge the presence of PAD and how severe it was. A summary judgment of the patient’s flow status was then made. Degree of vessel disease was scored and assessed using a DUS grading scheme. The scan protocol is given in the appendix.

## Results

A total of 258 patients were recruited from 15 different GPs across the North East of England from May 2015 to September 2016. Of these, 8 did not attend as invited for DUS imaging, leaving 250 patients (125 registered and 125 non-PAD registered) for analysis. Table 1 summarizes the baseline demographics for both PAD and non-PAD registered patients.

**Table 1.** Patient Demographics, Smoking Status, and PAD Registry Status.

	Total			Register PAD positive			Register PAD negative			*P*
Participants, n	250			125			125			—
DUS positive, n (%)	101 (40.4)			87 (69.6)			14 (11.2)			<.001
Age, y, mean ± SD	71.8 ± 8.6			72.5 ± 8.5			71.0 ± 8.6			.171
BMI, kg/m^2^, mean ± SD	27.3 ± 4.6			27.3 ± 5.5			27.3 ± 3.6			.971
Diabetes, n (%)	59 (23.6)			34 (27.2)			25 (20.0)			.233
HTN, n (%)	147 (58.8)			83 (66.4)			64 (51.2)			.017
Smoking status,^a^ n (%)	Never	Ex	Current	Never	Ex	Current	Never	Ex	Current	
	70 (28.0)	128 (51.2)	52 (20.8)	20 (16.0)	66 (52.8)	39 (31.2)	50 (40.0)	62 (49.6)	13 (10.4)	<.001
Male:female, n	156:94			79:46			77:48			.896
IHD, n (%)	59 (23.6)			47 (37.6)			12 (9.6)			<.001
Stroke, n (%)	18 (7.2)			14 (11.2)			4 (3.2)			.026
TIA, n (%)	23 (9.2)			18 (14.4)			5 (4.0)			.009
AF, n (%)	20 (8.0)			13 (10.4)			7 (5.6)			.244

Abbreviations: PAD, peripheral artery disease; BMI, body mass index; HTN, hypertension; IHD, ischemic heart disease; TIA, transient ischemic attack; AF, atrial fibrillation.

a “Current smoking status” was used as the comparator between register PAD positive and register PAD negative.

Of the 125 PAD registered patients recruited, 87 (69.6%) had DUS evidence of PAD in at least 1 leg when scanned. Of the 125 matched controls, 111 (88.8%) had no evidence of PAD on DUS imaging. The PAD register false positive rate was 30.4% and false negative rate 11.2% when assessed by DUS scanning. Across these 2 cohorts of patients, the PAD register had a sensitivity and specificity of 86% (95% CI 77%-92%) and 74% (95% CI 67%-81%), respectively. The positive predictive value, however, was 69.6% (95% CI 63%-75%) and negative predictive value 88.8% (95% CI 82%-92%). The overall diagnostic accuracy of the PAD register was 79.2% (95% CI 73%-84%).

In the PAD registry cohort, there was a significantly higher proportion of patients with ischemic heart disease (IHD) (*P* < .001), stroke (*P* = .03), transient ischemic attack (TIA) (*P* = .009), and hypertension (*P* = .017) compared with the control group (Table 1). No significant differences were found between the 2 groups with regard to age (*P* = .17), gender (*P* = .9), body mass index (*P* = .97), diabetes (*P* = .23), or atrial fibrillation (*P* = .24). Where PAD was confirmed on DUS, 65 (64.4 %) of the 101 patients positive for PAD had bilateral disease and 36 (35.6%) had unilateral disease.

## Discussion

The results of this case-control study suggest that GPs are good at detecting and registering PAD in their patients, with the PAD register demonstrating a sensitivity of 86%. There is however some room for improvement given the high rate of false positives (30.4%). While the false negative rate was much lower at 11.2%, this still represents a relatively large proportion of the population lacking complete risk factor control strategies. Transversely, the suggestion is many patients are potentially being exposed to the side effects of PAD-protective medication despite lacking true PAD. The reasons for misdiagnosing PAD are likely to be multifaceted but include the limitations of ABPI and clinical risk stratifying tools. Although previous work formally assessing these tools highlights their strengths as diagnostic adjuncts for the GP, this study depicts a potentially more applicable representation of how well they are used in the clinical environment.

Accurate and timely identification of PAD is important in prognostication and treatment planning.^11^ Detecting and screening for PAD in primary care has traditionally depended on ABPI and cardiovascular risk factor scoring systems.^6^ When used in isolation, these typically have low yields in identifying PAD in patients in primary care.^6^ Furthermore, a recent Cochrane review identified a lack of evidence in the accuracy of ABPI readings in diagnosing PAD in people with intermittent claudication.^12^ ABPI is widely lauded as an effective investigation comparable to angiographic imaging with a sensitivity of 95% and specificity of 99%.^13^ While there is some evidence to suggest that ABPI is effective at identifying the presence of PAD in asymptomatic at risk individuals in the hospital setting,^14^ ABPI has been demonstrated to be less effective at detecting lower grade stenosis, as may be present in asymptomatic patients in the community at risk of developing ischemic leg disease.^15^ Lewis et al^16^ improved the accuracy of ABPI by combining measurements with wave volume analysis, producing a 100% sensitive and 76% specific instruments when compared with DUS.^16^

There are a number of reasons that potentially explain the limitations of ABPI as a diagnostic tool in primary care.^8^ Despite efforts to standardize methodology, there still exists a lack of clear consensus in practice about which of the 2 brachial systolic values are used to calculate ABPI,^17^ the environmental factors such as practitioner room temperature and referral value thresholds.^8,18^ Previous investigators have explored exercise stress tests pre-ABPI as a means of improving diagnostic accuracy.^19^ However, this is often limited by the presence of bilateral disease and the most symptomatic limb limiting participation.^18^ Apart from the fact stress testing means more equipment and more time taken for an already lengthy investigation in time-pressured GP consultations.

ABPI is just one facet in assessing suspected PAD. Many practitioners use the presence of intermittent claudication symptoms. The shared clinical features and typical patient demographics of PAD and lumbar spinal stenosis mean there is potential for misdiagnosis.^20^ Studies by Han et al^20^ and Uesugi et al^21^ demonstrated that PAD is present in 4.1% and 6.7% of lumbar spinal stenosis patients, respectively, meaning a percentage of patients experience diagnostic overshadowing. Although this study would suggest more people are being diagnosed with PAD than have disease, it is worth noting that the absence of intermittent claudication may still falsely reassure the GP that PAD is absent. McDermott et al^22^ found IC was present in only 28.5% of patients with established PAD and absence of symptoms was more common in male, older, and diabetic patients.

PAD and its association to IHD and ischemic stroke is well described^23,24^ owing to a number of common risk factors.^25^ This may lead to presumptive diagnoses of PAD despite absent clinical or radiographic evidence. Clinical scoring systems that help guide diagnosis of related cardiovascular disease risk in primary care do not always correlate with presence of PAD, despite sharing many risk factors.^26^

Of all 258 patients invited to follow up, only 8 did not attend for DUS imaging. All control group patients were age- and sex-matched from the GP registers and were therefore likely to be representative of the general population. Equal numbers of cases and controls were assessed, appropriately matched for age and gender. All measurements, including duplex ultrasound scanning were performed within the practice to represent better the population under the care of the community physician. Although previous work has assessed different tools available to the practitioner in terms of sensitivity and specificity, this study evaluates how well current practice in primary care is establishing the burden of PAD in their practices.

### Limitations

GP registers were taken from practices listed under a research registry collaborative and selected based on willingness to cooperate with the study in order to allow for sufficient recruitment of patient numbers. All practices were within one region of the United Kingdom and may not be representative of national and international populations. Practices may have varied in their means of forming a diagnosis, for example, all practices studied had the capacity to measure ABPI, though how many PAD registered patients diagnosis was based on an ABPI value is not known.

### Implications for Research and Practice

In light of these limitations, new objective assessment tools for PAD in primary care have been evaluated. The use of photoplethysmography in determining pulsatile changes in the microcirculation of the skin at the toes is long standing.^27^ More sophisticated applications of this vascular optics technology can be used to identify changes in waveforms between healthy and arterially diseased legs.^28^ Further work performed as part of this study aims to assess its potential for the GP in providing a more sensitive and specific tool in assessing PAD, and without adding to an already time pressured consultation.

## Appendix

### Scan Protocol

- Bilateral scan of lower limb arteries to be performed on each subject
- Analysis by segments with final summary
- For each segment tick one box in each lettered group
- Comment on any factors affecting diagnosis
- Give your overall opinion on whether PAD is present and to what degree

Definition: A diagnosis of PAD will be made if there is any of

(a) At least one stenosis of >50%
(b) An occlusion
(c) General narrowing of such a degree that the flow is impeded and the waveform is damped in popliteal or distal segment.
